# Clinical Experience with Chitosan Matrix and Cultured Fibroblasts for Burns

**DOI:** 10.5195/cajgh.2014.157

**Published:** 2014-12-12

**Authors:** Gaziza Danlybayeva, Assylbek Zhylkibayev, Zhansaya Akhmadeyeva, Elena Belan, Zhanatay Ramazanov

**Affiliations:** 1 Laboratory of Stem Cells, National Center for Biotechnology, Astana, Kazakhstan; 2 Scientific Research Institute of Traumatology and Orthopedics, Astana, Kazakhstan

**Keywords:** burns, chitosan-pectin scaffold, fibroblast, epithelialization

## Abstract

**Introduction:**

Burns are an important public health challenge due to the frequency of getting burns in day-to-day life, occupational hazards, and catastrophes. Treatment of burns is complex and is associated with high morbidity and mortality. Duration and complexity of burn treatment require finding new ways of curing and rehabilitating burns. The result of burn treatment plays a significant role in post-traumatic status of a patient and his or her consequent adaptation in society. Chitosan is a natural safe non-toxic product compatible with human tissues, characterized by hydrosorbid, anticoagulant, antibacterial, and wound healing features. The study aims to show a clinical application of chitosan-pectin scaffold with cultured human skin fibroblasts in the treatment of deep burns.

**Methods:**

The substrate was prepared by dissolving 3% chitosan in 0.5N acetic acid, which was then mixed with 3% solution of pectin dissolved in distillated water. Chitosan film was formed in a Petri dish for 20–24 hours at 20–25 °C. After drying the film, cultured allogeneic fibroblasts (patent number RK-25091) were seeded on its surface.

**Results:**

The results from an in vitro culture study showed that human allogeneic fibroblasts could adhere well and grow on the selected scaffold with a typical morphology. During autodermoplasty surgery, cultured allogeneic fibroblasts were applied on granulating wounds of 9 patients with IIIA to IVB degree burns and limited donor resources. Wounds treated with the fibroblastseeded scaffold among all patients provided the highest level of re-epithelialization (day 5), in comparison to cell-free scaffold (day 7) and untreated surface of wounds (day 10).

**Conclusion:**

Our results indicate the potential use of chitosan for wound healing due to its allogenic fibroblast adherence to scaffolding as well as high epithelization. This warrants further studies on chitosan for use in wounds resulting from third and fourth degree burns.

